# Prehabilitation to reduce postoperative complications in frail and elderly patients with gastrointestinal cancer: a systematic review and meta-analysis

**DOI:** 10.3389/fonc.2026.1777929

**Published:** 2026-03-16

**Authors:** Xueni Liu, Liming Bao, Yanru Xie

**Affiliations:** Departnent of Cancer Center, Lishui Central Hospital (The Fifth Affiliated Hospital of Wenzhou Medical University), Lishui, Zhejiang, China

**Keywords:** aged, frailty, gastrointestinal neoplasms, meta-analysis, postoperative complications, prehabilitation

## Abstract

**Background:**

Elderly and frail patients with gastrointestinal cancer are at significantly increased risk of postoperative complications. The effectiveness of preoperative prehabilitation in this high-risk population requires synthesis of the existing evidence.

**Objective:**

To evaluate the impact of preoperative prehabilitation on the incidence of postoperative complications in elderly (≥60 years) and frail patients with gastrointestinal cancer through a systematic review and meta-analysis.

**Methods:**

We systematically searched PubMed, Embase, the Cochrane Library, and Web of Science from January 2020 to November 2025. Randomized controlled trials comparing prehabilitation with usual care were included. The primary outcome was the incidence of overall postoperative complications. Risk ratios were pooled using a random-effects model. Risk of bias was assessed using the ROB 2.0 tool. Subgroup analyses were conducted based on intervention duration (≥4 weeks vs. <4 weeks), surgery type (colorectal vs. other), and frailty status.

**Results:**

Nine randomized controlled trials involving 1027 patients were included. The meta-analysis showed that prehabilitation significantly reduced the risk of postoperative complications (risk ratio [RR] = 0.67, 95% confidence interval [CI]: 0.46 to 0.99, p = 0.04), with low heterogeneity among studies (I² = 24%). Subgroup analyses indicated a greater trend towards risk reduction in frail patients (RR = 0.53, 95% CI: 0.24 to 1.16), as well as in interventions lasting <4 weeks (RR = 0.54, 95% CI: 0.30-0.99) and in patients undergoing non-colorectal surgery (RR = 0.54, 95% CI: 0.30-0.99). However, differences between all subgroups were not statistically significant (p for interaction > 0.05).

**Conclusion:**

Current evidence suggests that preoperative prehabilitation reduces postoperative complication risk in elderly patients undergoing gastrointestinal cancer surgery. While the effect is clear in the overall population, the trend suggesting potentially greater benefit in specifically frail patients requires confirmation in future larger, specifically designed trials. Integrating prehabilitation into the clinical pathway for this high-risk population is warranted.

**Systematic Review Registration:**

https://www.crd.york.ac.uk/prospero/, identifier CRD420251275161.

## Introduction

Gastrointestinal cancers, defined in this review as malignancies of the esophagus, stomach, colorectum, liver, gallbladder, and pancreas, consistent with the GLOBOCAN and WHO classification systems represent a major global public health concern, with high incidence and mortality rates (1, 2). With the global aging population, the number of elderly patients undergoing curative or palliative surgery for gastrointestinal cancers continues to increase (3–5). Age-related decline in physiological reserve and multi-morbidity predispose older patients to higher risks of postoperative complications (6). This risk is particularly pronounced in those identified as “frail” by clinical tools such as the Fried phenotype (7). Consequently, identifying interventions to optimize preoperative status in older, and especially frail, surgical candidates is of paramount importance.

Prehabilitation, a multimodal intervention, has shown potential to reduce complications in general adult surgical populations (8). However, its efficacy when applied to older adults remains equivocal (9, 10). This may stem from the high heterogeneity within the elderly population and their differential response to standardized interventions. Notably, prehabilitation studies specifically designed for very old or frail patients remain scarce, and their findings have been inconsistent (11, 12). A synthesis of high-quality evidence regarding the effect of prehabilitation specifically for older patients undergoing gastrointestinal cancer surgery is currently lacking.

Therefore, this systematic review and meta-analysis aims to synthesize existing evidence to determine the overall impact of preoperative prehabilitation on postoperative complications in older (typically defined as ≥60 or ≥65 years) patients with gastrointestinal cancers. Recognizing frailty as a critical modifier of treatment effect, we also sought to examine the extent to which existing studies included patients explicitly assessed as frail, and to explore the trend of prehabilitation effect within this specific high-risk subgroup.

## Methods

### Study protocol and registration

This review was conducted and reported in accordance with the Preferred Reporting Items for Systematic Reviews and Meta-Analyses statement (13). The study protocol was registered on an international prospective register of systematic reviews.

### Search strategy

This research systematically searched four electronic databases: PubMed, Embase, the Cochrane Central Register of Controlled Trials (CENTRAL), and Web of Science Core Collection. The search timeframe was set from January 2020 to November 2025. The search strategies combined Medical Subject Headings and free-text terms, focusing on four key concepts: (1) gastrointestinal cancer, (2) prehabilitation, (3) elderly or frailty, and (4) postoperative complications. Searches were restricted to English-language publications. The full search strategies for each database are provided in the **Supplementary Material**. Additionally, reference lists of relevant systematic reviews were manually screened to identify potentially eligible studies. The protocol for this systematic review was registered in the International Prospective Register of Systematic Reviews (PROSPERO) under the registration number CRD420251275161.

### Study selection and eligibility criteria

Two reviewers independently screened studies against the pre-defined eligibility criteria. Initial screening was performed based on titles and abstracts, followed by a full-text assessment of potentially eligible articles. Discrepancies were resolved through discussion or adjudication by a third reviewer.

The eligibility criteria followed the PICO framework:

Population: Patients aged ≥60 years with gastrointestinal cancer scheduled for curative or palliative surgery.

Intervention: Structured prehabilitation programs comprising at least one component of exercise, nutritional support, or psychological intervention.

Comparison: Usual preoperative care.

Primary Outcome: Incidence of overall postoperative complications, defined by the Clavien-Dindo classification ≥ Grade II or similar explicit criteria.

Study Design: Randomized controlled trials (RCTs).

### Data extraction

Two reviewers independently extracted data using a standardized form. Extracted information included: first author, publication year, country, study design, sample size, patient baseline characteristics (including age, frailty status and assessment tool, details of the prehabilitation program, control group regimen, definition of postoperative complications, and number of events). In cases of missing or unclear data, corresponding authors were contacted via email for clarification.

### Methodological quality assessment

The methodological quality of included studies was independently assessed by two reviewers.

This research used the revised Cochrane Risk of Bias tool RoB 2.0 (14). This tool evaluates five domains: bias arising from the randomization process, deviations from intended interventions, missing outcome data, measurement of the outcome, and selection of the reported result. Each domain was judged as ‘low risk’, ‘some concerns’, or ‘high risk’. We assessed the risk of bias using the revised Cochrane Risk of Bias tool (ROB 2.0), entered the assessment data into Review Manager, and generated the ‘Risk of bias’ graph.

### Data synthesis and analysis

Prior to synthesis, the extracted data were prepared and checked for consistency. For studies reporting complications as a dichotomous outcome, data were directly used. For studies reporting only the mean number of complications per patient, these were analyzed separately as a continuous outcome. In cases where summary statistics were missing or unclear, we attempted to contact the corresponding authors via email for clarification. No data conversions were required for the primary meta-analysis as all included studies provided applicable risk ratio data or raw event counts.

All statistical analyses were performed using Review Manager 5.4 software. For the primary outcome, we pooled event numbers from the intervention and control groups of each study to calculate the risk ratio with its 95% confidence interval. As clinical and methodological heterogeneity were anticipated, a random-effects model was used for the meta-analysis. Heterogeneity among studies was assessed using the I² statistic and Cochrane’s Q test. An I² value > 50% or a Q test p-value < 0.10 was considered indicative of substantial heterogeneity.

The following analyses were planned:

1. Primary analysis: A pooled estimate of the overall effect of prehabilitation on postoperative complications across all included studies.

2. Subgroup analyses: To explore potential sources of heterogeneity and assess effect modification, we conducted three pre-specified subgroup analyses:(1) Intervention Duration: Studies with prehabilitation programs lasting ≥4 weeks;

(2) Cancer Type: Studies focusing exclusively on colorectal cancer;

(3) Population Risk Profile: Studies that specifically enrolled and assessed patients as frail using validated tools.

3. Sensitivity analysis: The robustness of the pooled results was tested by sequentially removing each study.

4. Assessment of publication bias: Visual inspection of funnel plot asymmetry was planned if ten or more studies were included in the meta-analysis.

## Results

### Study selection process

**Figure 1:**
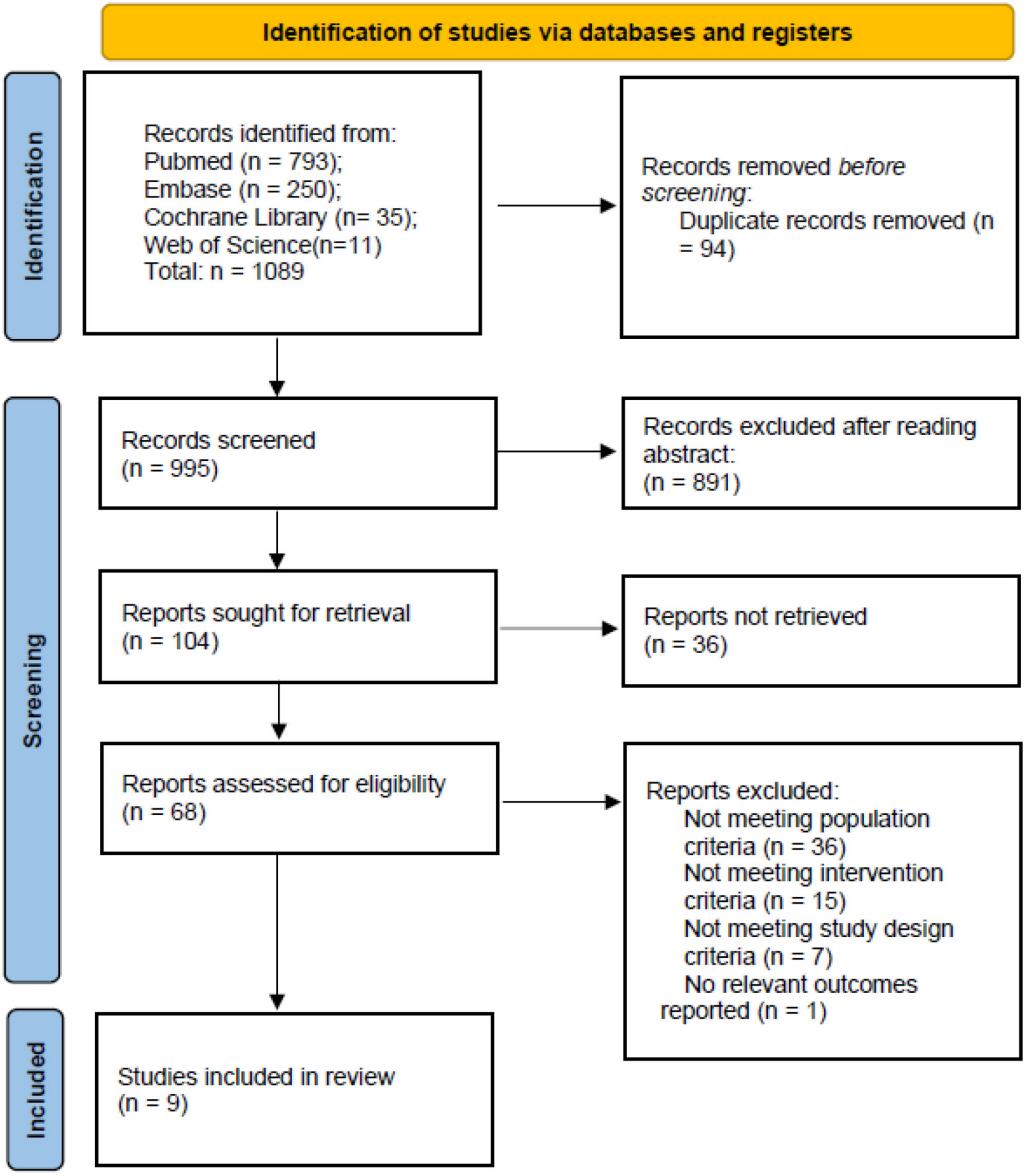
PRISMA flow diagram.

A total of 1089 records were identified through systematic searches of four databases. After removing 94 duplicates, 995 records remained for title and abstract screening. During this phase, 891 records were excluded as clearly irrelevant. Full texts were retrieved for the remaining 104 articles. Of these, 36 articles could not be retrieved despite attempts through interlibrary loan and direct contact with corresponding authors. The main reasons for non-retrieval included: publication only as conference abstracts without subsequent full-text publication (n = 19), retracted or withdrawn status (n = 5), non-English language without available translation (n = 7), and inability to obtain full text via institutional access or author contact (n = 5). Following a detailed assessment of the 68 available full-text articles, 9 studies met the eligibility criteria and were included in the systematic review and meta-analysis. The detailed screening process and reasons for exclusion are presented in **Figure 1** (PRISMA Flow Diagram).

### Characteristics of included studies

The 9 included studies (11, 12, 15–21) were all randomized controlled trials (RCTs) published between 2020–2025 and involved a total of 1027 participants. The study populations primarily targeted colorectal cancer patients (5 studies). All studies targeted older patients (mean age ≥65 years or explicitly used “elderly” as an inclusion criterion). Six studies specifically enrolled and assessed patients as frail using validated tools. The prehabilitation programs were predominantly multimodal (8 studies), with intervention durations ranging from almost 2 (mean 10.52 days) to 6 weeks. All studies reported the incidence of overall postoperative complications as a primary or secondary outcome. Detailed characteristics of the included studies are presented in **Table 1**.

**Table 1:** Characteristics of included studies.

Authors	Year	Study type	Population	Sample size	Use of specific tool for frailty assessment	Prehabilitation program	Usual Care program	Intervention duration	Primary outcome	Secondary outcomes
Molenaar et al. (20)	2023	RCT	Patients with nonmetastasized colorectal cancer surgery	251 (ITT analysis)	Not reported	4-week in-hospital supervised multimodal prehabilitation (high-intensity interval training, nutritional intervention, psychological support, smoking cessation)	Standard perioperative care following ERAS pathway	4 weeks (preoperatively)	CCI at 30 days & 6-minute walking distance at 4 weeks postop	Severity of complications, functional recovery, nutritional & mental status, quality of life, etc.
Jiang et al. (17)	2025	RCT	Patients undergoing endoscopic submucosal dissection (ESD)	100 (ITT analysis)	Yes, Fried Frailty Index	4-week multimodal prehabilitation (supervised aerobic/resistance training, individualized nutrition, psychological support)	Standard preoperative education, no structured exercise or nutrition program	4 weeks (preoperatively)	Change in 6-minute walk distance at 1 month post-ESD	Grip strength, quality of life, psychological status, complications, length of stay, readmissions, etc.
Lv et al. (19)	2024	RCT	Elderly undergoing gastrointestinal surgery	154 (ITT analysis)	Yes, FRAIL scale	Low-intensity perioperative rehabilitation exercise (initiated after admission until 30 days postop), including limb & breathing exercises, abdominal massage, muscle strengthening, etc.	Standard perioperative care (preoperative education, nutritional care, early ambulation, etc.)	From admission until 30 days post-surgery	Comprehensive Complications Index at 30 days postop	Complication rates, readmission, satisfaction, quality of life, psychological status, etc.
Bojesen et al. (11)	2023	RCT	Colorectal cancer patients with WHO performance status I/II	40 (randomized), 36 (analyzed)	Yes (Geriatric 8 score)	Supervised HIIT + nutritional supplements + medical optimization	Standard care + ERAS	4 weeks	Quality of recovery in first 3 postoperative days (QoR-15)	Physical fitness, physical function, nutritional status, bloodwork, postoperative complications
Yuan & Chu (21)	2025	RCT	Patients scheduled for pancreaticoduodenectomy	88	Not Reported	Aerobic exercise + breathing training + nutritional guidance + psychological support	Standard preoperative care	Mean 10.52 days	6-minute walk test distance	Postoperative complications, quality of life, HADS scores
López-Rodríguez-Arias et al. (18)	2021	RCT	Colorectal cancer surgery patients (during COVID-19)	20	Yes (Canadian Study of Health and Aging Clinical Frailty Scale)	Home exercise videos + high-protein supplements + relaxation exercises	ERAS without prehabilitation	30 days before surgery	Changes in lean mass and fat mass (bioelectrical impedance)	Length of stay, postoperative complications, HADS scores
Fulop et al. (16)	2021	RCT	Patients undergoing colorectal surgery	149 (intention-to-treat)	Not Reported	Trimodal (exercise, nutrition, psychological)	ERAS + nutritional support if needed	3–6 weeks	6-minute walking distance, respiratory function, quality of life, anxiety and depression scores	Length of stay, postoperative complications, mortality, time to adjuvant chemotherapy
Carli et al. (12)	2020	RCT	Frail patients undergoing colorectal cancer resection	110 (intention-to-treat)	Yes, Fried Frailty Index	Multimodal (exercise, nutrition, psychological)	Postoperative rehabilitation (same intervention but post-surgery) + ERAS	4 weeks	30-day Comprehensive Complication Index (CCI)	Overall complications, severe complications, length of stay, readmissions, walking capacity, patient-reported outcomes
Chen et al. (15)	2024	RCT	Frail elderly patients undergoing gastric cancer surgery	115 (final analysis)	Yes, Fried Frailty Index	Multimodal (aerobic & resistance exercise, respiratory training, nutrition, psychological)	Usual clinical care + ERAS	3 weeks	30-day Comprehensive Complication Index (CCI)	6-minute walking distance, length of stay, readmissions, quality of life, psychological status, pulmonary function

### Methodological quality assessment

**Figure 2:**
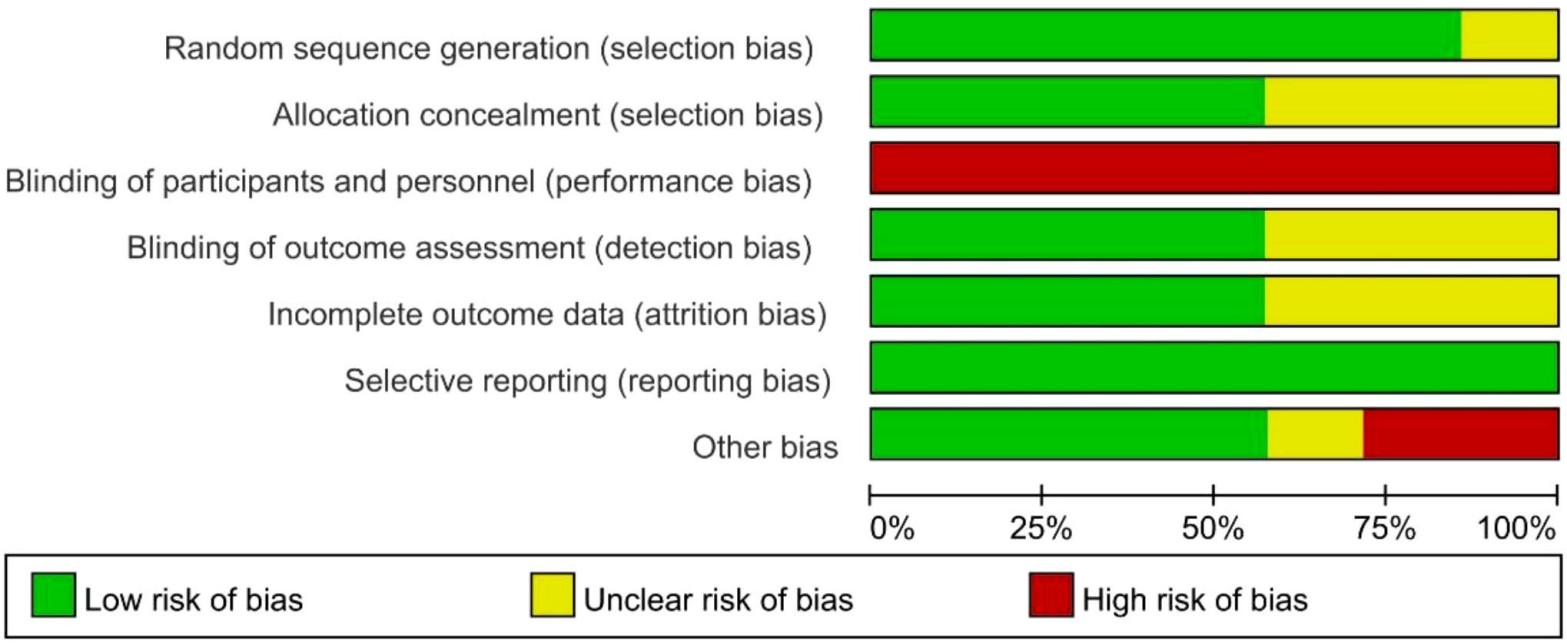
Risk of bias graph.

All 9 included studies were RCTs. The overall methodological quality is high, with most studies showing low risk across bias domains, a few with unclear risk, and minimal high risk of bias. Detailed results of the risk of bias assessment are presented in **Figure 2** (Risk of Bias Summary).

### Meta-analysis results

#### Postoperative complications

A total of seven RCTs reporting dichotomous outcome were included in the primary meta-analysis comparing prehabilitation with usual care (or usual care plus postoperative rehabilitation) on postoperative complications in older and frail patients. The pooled result demonstrated a statistically significant reduction in complication risk favoring the prehabilitation group. The overall risk ratio was RR = 0.67 (95% CI 0.46 to 0.99, p = 0.04).

**Figure 3:**
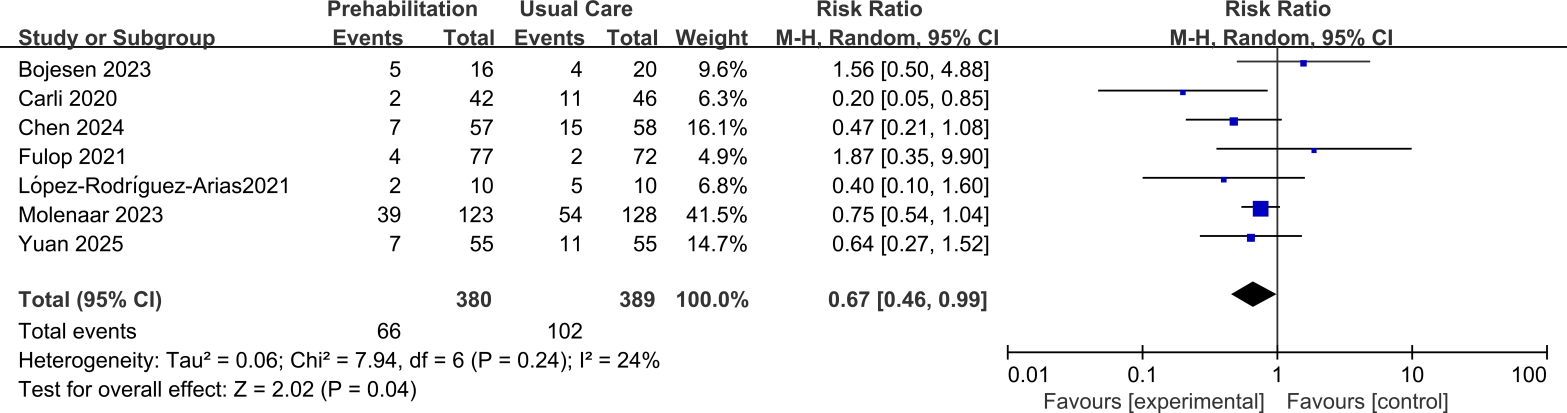
Forest plot of comparison between prehabilitation and usual care 1.

Statistical heterogeneity across studies was low-to-moderate (I² = 24%, Tau² = 0.06, P = 0.24, Chi (2) = 7.94), indicating consistency in the direction of effect. The visual inspection of the forest plot did not identify any substantial outliers, supporting the robustness of the pooled estimate, as shown in **Figure 3**.

**Figure 4:**
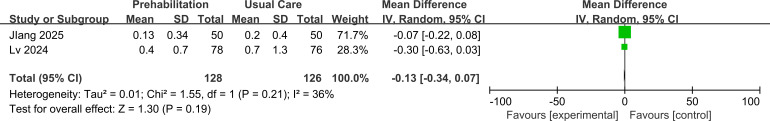
Forest plot of comparison between prehabilitation and usual care 2.

Two studies reported the mean number of complications per patient as a continuous outcome and were analyzed separately. The pooled mean difference was MD = -0.13 (95% CI -0.34 to 0.07, p = 0.19). Although this result did not reach statistical significance, the point estimate suggests a trend towards a lower complication burden (fewer complications per patient) in the prehabilitation group. Heterogeneity was moderate (I² = 36%, Tau² = 0.01, P = 0.21, Chi^2^ = 1.55), as shown in **Figure 4**.

### Subgroup analyses

**Figure 5:**
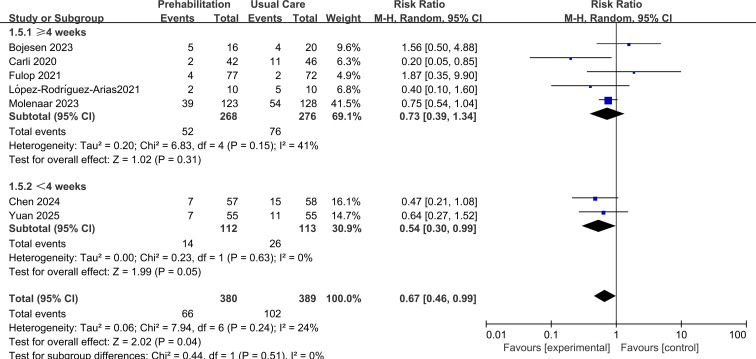
Forest plot of subgroup1: intervention duration.

In a subgroup analysis by intervention duration, a non-significant reduction in complications was observed for programs lasting ≥4 weeks (RR = 0.73, 95% CI 0.39 to 1.34, p=0.31; *I*²=41%), whereas programs of <4 weeks showed a statistically borderline benefit (RR = 0.54, 95% CI 0.30 to 0.99, p=0.04; *I*²=0%). However, the test for subgroup differences was not significant (p=0.51), indicating no clear evidence of an effect modification by intervention length, as shown in **Figure 5**.

**Figure 6:**
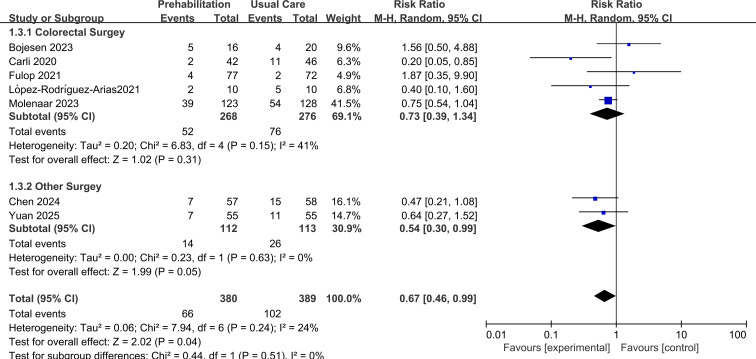
Forest plot of subgroup2: surgical type.

A subgroup analysis based on surgery type suggested a potential differential effect. The benefit of prehabilitation appeared more pronounced, with borderline significance, in patients undergoing “other” (non-colorectal) surgeries (RR = 0.54, 95% CI 0.30 to 0.99, p=0.05) compared to those undergoing colorectal surgery (RR = 0.73, 95% CI 0.39 to 1.34, p=0.31). However, the test for subgroup differences was not significant (p=0.51), precluding a definitive conclusion regarding effect modification by surgery type, as shown in **Figure 6**.

**Figure 7:**
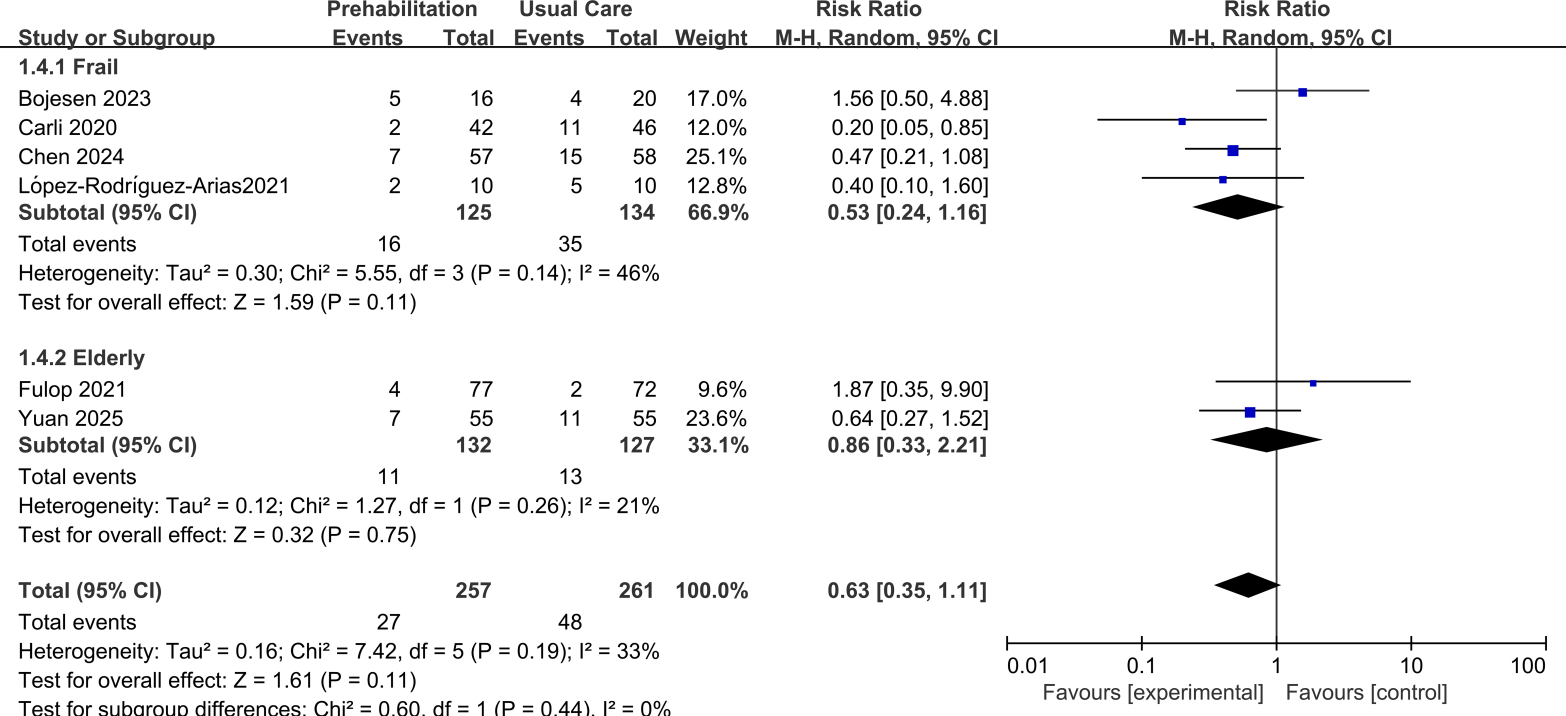
Forest plot of subgroup3: frail vs elderly.

Subgroup analysis by frailty status showed a non-significant trend towards benefit in frail patients (RR = 0.53, 95% CI 0.24 to 1.16, p=0.11), with no significant effect observed in studies of non-frail elderly patients (RR = 0.86, 95% CI 0.33 to 2.21, p=0.75). The test for subgroup differences was not significant (p=0.44), as shown in **Figure 7**.

## Discussion

### Main finding

This systematic review and meta-analysis in older and frail patients undergoing gastrointestinal cancer surgery indicates that preoperative prehabilitation significantly reduces the overall risk of postoperative complications. This finding aligns with the established benefits of prehabilitation observed in general surgical populations (8). It is noteworthy that while the effect size for the primary outcome reached statistical significance, the upper limit of its confidence interval approached the line of null effect (RR = 0.67, 95%CI 0.46-0.99), suggesting a degree of caution is warranted when interpreting its clinical magnitude. Importantly, subgroup analyses revealed several clinically informative trends: a greater, though non-significant, trend towards risk reduction was observed in frail patients, and programs shorter than 4 weeks showed comparable potential benefit to longer ones.

### Result interpretation and mechanism exploration

Potential Benefit in Frail Populations: This review identified a clear trend towards benefit (RR = 0.53) from prehabilitation in patients explicitly diagnosed as frail, with a point estimate superior to the overall population. This is physiologically plausible, as frail individuals with diminished physiological reserve and higher vulnerability might obtain a greater “marginal gain” from structured interventions aimed at enhancing functional capacity. However, the underpowered nature of this subgroup analysis underscores the critical paucity of high-quality evidence. This finding strongly advocates for the routine inclusion and standardized reporting of frailty status in future trials to validate this hypothesis (22, 23).

Unexpected Finding on Intervention Duration: Contrary to the initial hypothesis, prehabilitation programs shorter than 4 weeks showed a comparable or even more favorable point estimate than longer programs. This may challenge the simplistic notion that “longer is better.” A plausible explanation is that for older or frail patients, a short-term, high-adherence intervention may be more feasible and sustainable than a complex, prolonged program, leading to a better net effect in practice. This suggests that “intervention intensity” and “patient engagement” might be more critical success factors than mere “duration” alone (24).

Effect by Surgery Type: The more pronounced trend towards benefit in patients undergoing non-colorectal surgeries (e.g., gastric, esophageal) may reflect the inherently higher physiological stress and complication risks associated with these upper GI procedures, allowing the physiological optimization from prehabilitation to yield a greater relative benefit. This finding warrants further exploration in larger future studies.

### Comparison with existing literature

The findings of this review align with the direction of conclusions from existing systematic reviews on the role of prehabilitation in patients undergoing abdominal cancer surgery. For instance, a 2017 systematic review noted that prehabilitation programs in abdominal cancer surgery showed potential for improving postoperative outcomes (25). Our analysis demonstrated a clear risk reduction (RR = 0.67) in an older, higher-risk population undergoing gastrointestinal cancer surgery, providing new evidence for the effectiveness of prehabilitation in a more specific cohort. Furthermore, the emergence of large-scale randomized controlled trial protocols in recent years, such as the PRIORITY trial, reflects ongoing efforts to build high-level evidence in this field (26). The findings of this review resonate with conclusions from recent randomized controlled trials focusing on prehabilitation in specific high-risk populations or cancer types. For instance, the pioneering work by Barberan-Garcia et al. demonstrated that personalized prehabilitation for high-risk patients undergoing major abdominal surgery significantly reduced severe postoperative complications (27). This study, alongside our findings, reinforces a central tenet: tailoring interventions based on patient risk profiles (such as frailty status in our analysis) is crucial. Similarly, the recent trial by Bausys et al. confirmed that a home-based prehabilitation program for patients undergoing gastric cancer surgery could be safely implemented and reduced complications (28). This aligns with the trend observed in our subgroup analysis, where a potentially greater benefit was suggested for patients undergoing non-colorectal (predominantly upper gastrointestinal) surgeries. Collectively, these high-quality trials indicate that the value of prehabilitation is evolving from “general applicability” towards “precision optimization,” where targeting specific surgical types and high-risk phenotypes (e.g., frailty) may yield superior outcomes.

The potential benefit observed for shorter-duration prehabilitation (<4 weeks) in our study aligns with conclusions from recent feasibility and implementation research focusing on frail older patients. A feasibility study by Bojesen et al. confirmed that a short-term, intensive, multimodal prehabilitation program is feasible and achieves good patient engagement in high-risk frail patients with colon cancer (29). This provides practical support for our finding that for frail patients with limited physiological reserve, a shorter, more condensed intervention may be more operationally viable than a protracted program, thereby ensuring the quality of intervention completion. Furthermore, a recent qualitative study with healthcare professionals identified multiple barriers and facilitators to implementing prehabilitation in frail older patients (30). This research indirectly suggests that a focused, shorter-duration prehabilitation program may face fewer implementation barriers and be more readily integrated into time-constrained preoperative pathways compared to longer programs that demand greater resources, time, and patient endurance. Consequently, “intervention intensity” and “completion fidelity” may be more critical determinants of success than the pursuit of duration alone.

## Strengths and limitations

### Strengths

This review adhered strictly to PRISMA guidelines and was prospectively registered. We conducted comprehensive searches and employed the up-to-date ROB 2.0 tool for risk of bias assessment. It is particularly noteworthy that we made an attempt to quantitatively synthesize evidence from prehabilitation studies targeting frail patients, providing preliminary insights into this critical area despite the limited number.

### Limitations

Several limitations should be acknowledged. First, the overall sample size of included studies remains modest, and the wide confidence interval for the primary outcome limits the precision of the conclusion. Second, despite conducting subgroup analyses, the number of studies within each subgroup was small, especially the frail patient subgroup comprising only four studies, resulting in underpowered analyses that preclude definitive conclusions. Third, heterogeneity existed across studies regarding the specific components and intensity of prehabilitation programs, as well as the definition and measurement of complications, introducing clinical diversity. Finally, meaningful assessment of publication bias was not feasible due to the inclusion of fewer than 10 studies.

## Clinical implications and future research directions

The findings of this review provide supporting evidence for considering the implementation of preoperative prehabilitation in older patients undergoing gastrointestinal cancer surgery. For clinicians, initiating a structured prehabilitation program, even if time is limited (e.g., to less than 4 weeks), may be beneficial. Decisions should be informed by an assessment of individual patient factors, including frailty status.

Future research should prioritize addressing the following gaps: (1) Conducting large-scale, multicenter RCTs specifically targeting patients assessed as frail using standardized tools to determine the efficacy and optimal regimen of prehabilitation in this population; (2) Exploring and optimizing “low-dose, high-intensity” or home-based prehabilitation models suitable for the old and frail to improve feasibility and adherence; (3) Routinely integrating comprehensive geriatric assessment into study designs and reporting relevant subgroup analyses to identify patient subgroups most likely to benefit.

## Conclusion

In conclusion, existing evidence suggests that preoperative prehabilitation can reduce the risk of postoperative complications in older patients undergoing gastrointestinal cancer surgery. Although statistically significant, the precision of this effect warrants confirmation from more research. Frail patients may derive greater benefit, a hypothesis that urgently requires testing in specifically designed, high-quality studies. Integrating preoperative prehabilitation into the clinical pathway for geriatric oncologic surgery, coupled with individualized risk assessment, represents a promising strategy.

## Data Availability

The original contributions presented in the study are included in the article/**Supplementary Material**. Further inquiries can be directed to the corresponding author.
