# Supplementary material for: Prehabilitation to reduce postoperative complications in frail and elderly patients with gastrointestinal cancer: a systematic review and meta-analysis
Source: Front Oncol. 2026 Mar 16;16:1777929. doi: 10.3389/fonc.2026.1777929 (PMC13033503; doi:10.3389/fonc.2026.1777929)
Supplement: Supplementary file 1 [file Supplementaryfile1.docx]

**Supplementary Material: Search Strategy**

**Last search date:** 22 December 2025

**PubMed**

**Number of records retrieved:** 793

("Gastrointestinal Neoplasms"[Mesh] OR "Colorectal Neoplasms"[Mesh] OR "Stomach Neoplasms"[Mesh] OR "Esophageal Neoplasms"[Mesh] OR "Pancreatic Neoplasms"[Mesh] OR "Liver Neoplasms"[Mesh])

OR

((gastrointestinal OR colorectal OR colon OR rectal OR stomach OR gastric OR esophagus OR oesophagus OR pancreatic OR liver)

AND (neoplas* OR cancer* OR carcinoma* OR adenocarcinoma* OR tumor* OR tumour* OR malignan*))

AND

("Preoperative Care"[Mesh] OR "Prehabilitation"[Mesh] OR "Exercise Therapy"[Mesh] OR "Nutrition Therapy"[Mesh])

OR

(prehab* OR pre-hab* OR prehabilitation OR preoperative rehabilitation OR pre-surgical rehabilitation OR preoperative training OR preoperative exercise* OR preoperative nutrition* OR (enhanced recovery AND preoperative))

AND

("Frailty"[Mesh] OR "Frail Elderly"[Mesh] OR "Aged"[Mesh])

OR

(frail* OR elder* OR geriatric OR "older adult*" OR "older patient*" OR senior OR "advanced age")

AND

("Postoperative Complications"[Mesh])

OR

(postoperative complication* OR surgical complication* OR post-surgical complication*)

AND

(english[la])

**Embase**

**Number of records retrieved:** 250

#1 gastrointestinal cancer OR colorectal cancer OR gastric cancer OR esophageal cancer OR pancreatic cancer OR liver cancer

#2 prehabilitation OR prehab*

#3 frailty OR elderly OR aged OR geriatric

#4 postoperative complication OR surgical complication

#1 AND #2 AND #3 AND #4

#5 #1 AND #2 AND #3 AND #4 AND publication year: 2020–2026

**Cochrane Library**

**Number of records retrieved:** 35

((gastrointestinal OR colorectal OR colon OR rectal OR stomach OR gastric OR esophagus OR oesophagus OR pancreatic OR liver) NEAR/3

(cancer OR neoplas* OR carcinoma* OR adenocarcinoma* OR tumor* OR tumour*))

AND

(prehab*)

AND

(frail* OR elder* OR geriatric OR "older adult" OR "older patient" OR senior)

AND

("postoperative complication" OR "surgical complication" OR "post-surgical complication")

**Web of Science**

**Number of records retrieved:** 11

((gastrointestinal OR colorectal OR colon OR rectal OR stomach OR gastric OR esophagus OR oesophagus OR pancreatic OR liver) NEAR/2

(cancer OR neoplas* OR carcinoma* OR adenocarcinoma*))

AND

(prehab*)

AND

(frail* OR elder* OR geriatric OR "older adult" OR "older patient")

AND

("postoperative complication" OR "surgical complication")
